# Red foxes harbor two genetically distinct, spatially separated *Echinococcus multilocularis* clusters in Brandenburg, Germany

**DOI:** 10.1186/s13071-021-05038-0

**Published:** 2021-10-14

**Authors:** Mandy Herzig, Pavlo Maksimov, Christoph Staubach, Thomas Romig, Jenny Knapp, Bruno Gottstein, Franz J. Conraths

**Affiliations:** 1 Friedrich-Loeffler-Institut, Federal Research Institute for Animal Health, Institute of Epidemiology, Südufer 10, 17493 Greifswald-Insel Riems, Germany; 2 Universität Hohenheim, Institut Für Biologie, Fachgebiet Parasitologie, Emil-Wolff-Straße 34, 70599 Stuttgart, Germany; 3 UMR CNRS 6249 Laboratoire Chrono-Environnement, Université Bourgogne Franche-Comté, 16 Route de Gray, 25030 Besançon, France; 4 Department of Parasitology-Mycology, National Reference Centre for Echinococcoses, University Hospital of Besançon, 25030 Besançon, France; 5 Institute for Infectious Diseases, Faculty of Medicine, University of Berne, 3001 Berne, Switzerland

**Keywords:** *Echinococcus multilocularis*, Fox, Genotypes, Spatial distribution, Germany, EmsB, Microsatellite, Short tandem repeat, Cluster

## Abstract

**Background:**

Alveolar echinococcosis (AE) is a clinically serious zoonosis caused by the fox tapeworm *Echinococcus* *multilocularis.* We studied the diversity and the distribution of genotypes of *E. multilocularis* isolated from foxes in Brandenburg, Germany, and in comparison to a hunting ground in North Rhine-Westphalia.

**Methods:**

*Echinococcus multilocularis* specimens from 101 foxes, 91 derived from Brandenburg and 10 derived from North Rhine-Westphalia, were examined. To detect potential mixed infections with different genotypes of *E. multilocularis*, five worms per fox were analyzed. For genotyping, three mitochondrial markers, namely cytochrome c oxidase subunit 1 (*Cox1*), NADH dehydrogenase subunit 1 (*Nad1*), and ATP synthase subunit 6 (*ATP6*), and the nuclear microsatellite marker EmsB were used. To identify nucleotide polymorphisms, the mitochondrial markers were sequenced and the data were compared, including with published sequences from other regions. EmsB fragment length profiles were determined and confirmed by Kohonen network analysis and grouping of Sammon’s nonlinear mapping with *k*-means clustering. The spatial distribution of genotypes was analyzed by SaTScan for the EmsB profiles found in Brandenburg.

**Results:**

With both the mitochondrial makers and the EmsB microsatellite fragment length profile analyses, mixed infections with different *E. multilocularis* genotypes were detected in foxes from Brandenburg and North Rhine-Westphalia. Genotyping using the mitochondrial markers showed that the examined parasite specimens belong to the European haplotype of *E. multilocularis*, but a detailed spatial analysis was not possible due to the limited heterogeneity of these markers in the parasite population. Four (D, E, G, and H) out of the five EmsB profiles described in Europe so far were detected in the samples from Brandenburg and North Rhine-Westphalia. The EmsB profile G was the most common. A spatial cluster of the *E.* *multilocularis* genotype with the EmsB profile G was found in northeastern Brandenburg, and a cluster of profile D was found in southern parts of this state.

**Conclusions:**

Genotyping of *E. multilocularis* showed that individual foxes may harbor different genotypes of the parasite. EmsB profiles allowed the identification of spatial clusters, which may help in understanding the distribution and spread of the infection in wildlife, and in relatively small endemic areas.

**Graphical Abstract:**

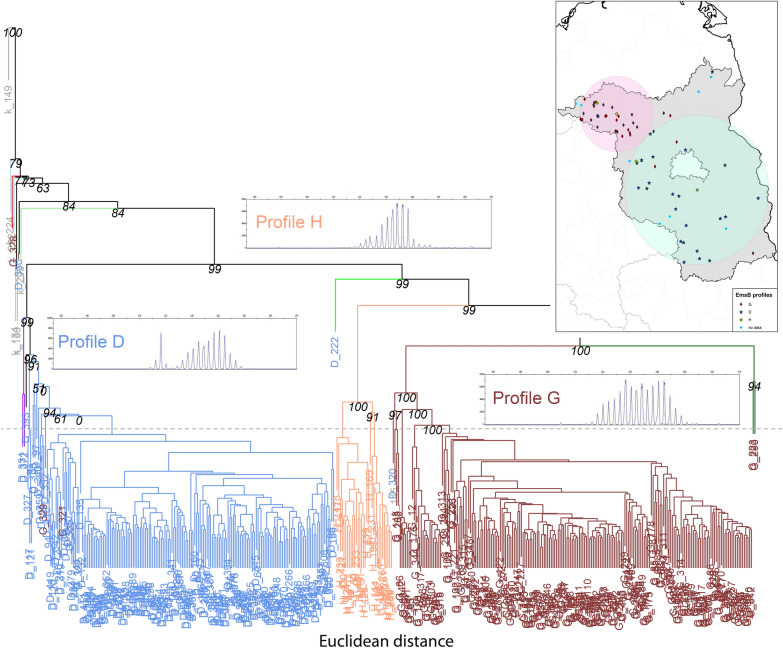

**Supplementary Information:**

The online version contains supplementary material available at 10.1186/s13071-021-05038-0.

## Background

*Echinococcus multilocularis* is regarded as the cause of one of the most important parasitic zoonoses in Europe (for reviews see Eckert et al. [1]; Vuitton et al. [2]; Wen et al. [3]). Human infections can result in severe disease, characterized as alveolar echinococcosis (AE), which is usually lethal if left untreated (for a review see Vuitton et al. [2]).

*Echinococcus multilocularis* is present in the Northern Hemisphere, where infections occur in Europe, Asia, and parts of North America. Studies conducted in Europe suggest that the area where *E. multilocularis* occurs is larger than previously assumed. In the late 1980s, only Austria, France, Germany, and Switzerland were reported as affected countries. By 2000, it was clear that the parasite was present in at least 11 countries [1]. Oksanen and colleagues [4] conducted a systematic review in 2016 and found that *E. multilocularis* had been detected in more than 20 countries in Europe at that point. Currently, the only major European countries without records of *E. multilocularis* detection are the United Kingdom, Ireland, Finland, Portugal, Spain, Bosnia–Herzegovina, Montenegro, and North Macedonia [5]. Moreover, the United Kingdom, Finland, Ireland, and Malta are officially regarded as non-endemic based on surveillance [4].

To elucidate the spatial and temporal dynamics in the distribution of *E. multilocularis*, genetic studies have been undertaken. While genotypes of *E. granulosus *sensu lato (s.l.) have been defined and can be more or less assigned to specific definitive host/intermediate host combinations, there is an ongoing debate on the question of whether different genotypes of *E. multilocularis* can be distinguished and, if so, how they can be characterized [6]. The first attempts of genotyping *E. multilocularis* used the mitochondrial marker located in the cytochrome c oxidase subunit 1 (*Cox1*) gene [7, 8]. Due to the small number of detected nucleotide differences, a division into only two geographically separate genotypes was established, i.e. M1, including isolates from China, Alaska, and North America, and M2, comprising isolates from Europe. This division into two major genotypes and their geographical distribution was confirmed by Rinder et al. [9] using a nuclear 18S rRNA marker. Haag and colleagues [10] found a different spatial distribution of two parasite populations identified with a homeobox gene marker. With this marker, one population contained samples that were distributed worldwide, and the other one consisted of samples detected on St. Lawrence Island, Alaska. Later, using a combination of mitochondrial markers (*cox1, nad2,* and *cob*), four clades were recognized which could be approximately correlated with their geographical origin in Europe, Asia, and North America, plus a divergent variant from Mongolia [11]. These clades are still widely cited, although it became apparent in the meantime that this geographical correlation is blurred since, for example, the “Asian” clade also occurs in Europe [12]. Generally, due to the low degree of variation in the mitochondrial sequences, these markers are considered to be useful on continental scales, but they seem unsatisfactory in their fine resolution of the genetic structure of this parasite, such as within a region of Europe. Therefore, the need to develop new tools for genotyping *E. multilocularis* was recognized some time ago.

In 1996, Bretagne and colleagues reported on the classification of *E. multilocularis* using microsatellites [13]. This work confirmed the differences between the geographical areas of North America/Alaska and Europe and indicated a low variability within the species *E. multilocularis* compared to *E. granulosus* s.l.. Ten years later, Bart and colleagues [14] discovered a new microsatellite marker, called EmsB. With this marker, it was possible not only to divide the isolates into the North American/Alaskan and European groups, but also to identify different genotypes within the groups. The motif of the marker is a tandem repetition of (CA)*m* and (GA)*n* followed by a GGTG sequence section followed by a repetition of (GA)*o*, where *m*, *n*, and *o* represent the number of repeats [14]. Different fragment lengths in the range of 209 to 247 base pairs (bp) of these EmsB amplicons can therefore be detected. The corresponding genotypes are thus characterized by a certain frequency of repeats at a defined fragment length in this range. Valot and coworkers further found that approximately 40 copies of the microsatellite were present on chromosome 5 [15].

The EmsB marker has been widely used for genotyping, especially in Europe. With the help of this marker, a greater diversity of *E. multilocularis* genotypes has been observed. Initially, 25 genotypes were defined in a global panel, 20 of which could be found in Europe [14]. Knapp and colleagues [16] divided the parasites from Europe into five main genotypes, which they called EmsB profiles (D, E, F, G, and H). The profile D can be further divided into four closely related subgroups [16]. With the typing of more *E. multilocularis* isolates, the number of profiles detected in Europe grew to 32 [17]. In recent years, EmsB profiles have been used for genotyping *E. multilocularis* isolates from a variety of countries including France [18–20], Switzerland [21], Northern Italy [22], Svalbard (Norway) [23], Kyrgyzstan [24], Estonia [25], Poland [26], Denmark, Sweden [27], Russia, Turkey, and many other European countries [28].

Until the late 1980s, only the southwestern part of Germany, i.e. Baden-Wuerttemberg and the region of Swabia in Bavaria, was regarded as endemic for *E. multilocularis* [1]. However, investigations in Rhineland-Palatinate in 1982 and 1983 demonstrated the presence of *E. multilocularis*-infected foxes in this federal state [29]. The parasite has also been reported in foxes in Hesse, North Rhine-Westphalia, and Lower Saxony since at least the 1980s [30]. By 1999, *E. multilocularis* had been detected in all German federal states except for the cities with federal state status, i.e. Berlin, Bremen, and Hamburg. At this time, the eastern part of Brandenburg seemed to be almost free from *E. multilocularis*. While the parasite was also detected in this region in the following years, the prevalence was lower than in the northwestern part of this federal state [31]. For Brandenburg, where most of the samples analyzed in the present study were obtained, a spatial and temporal spread of *E. multilocularis* in the fox population was demonstrated after 1992 [32].

Because of intensive monitoring and a trial to control *E. multilocularis* in foxes, the epidemiological situation in this German federal state has been was well characterized [32–36]. The first cases of *E. multilocularis* in foxes were observed in northwestern Brandenburg in 1991 [36]. A few years later, the formation of an endemic area in this region was reported [32].

Because of the dynamic situation in Brandenburg, we selected this region to study the potential of three mitochondrial markers, i.e. part of the cytochrome c oxidase subunit 1 (*Cox1*), NADH dehydrogenase subunit 1 (*Nad1*), and ATP synthase subunit 6 (*ATP6*) genes, and the nuclear EmsB microsatellite marker, to analyze the spatial distribution of *E. multilocularis* at the regional level. The aim was the fine-tuning of available tools for use in spatio-temporal molecular epidemiological analysis to understand the spread and genetic diversity of this zoonotic parasite in a dynamic endemic region.

## Methods

### Study area

**Fig. 1:**
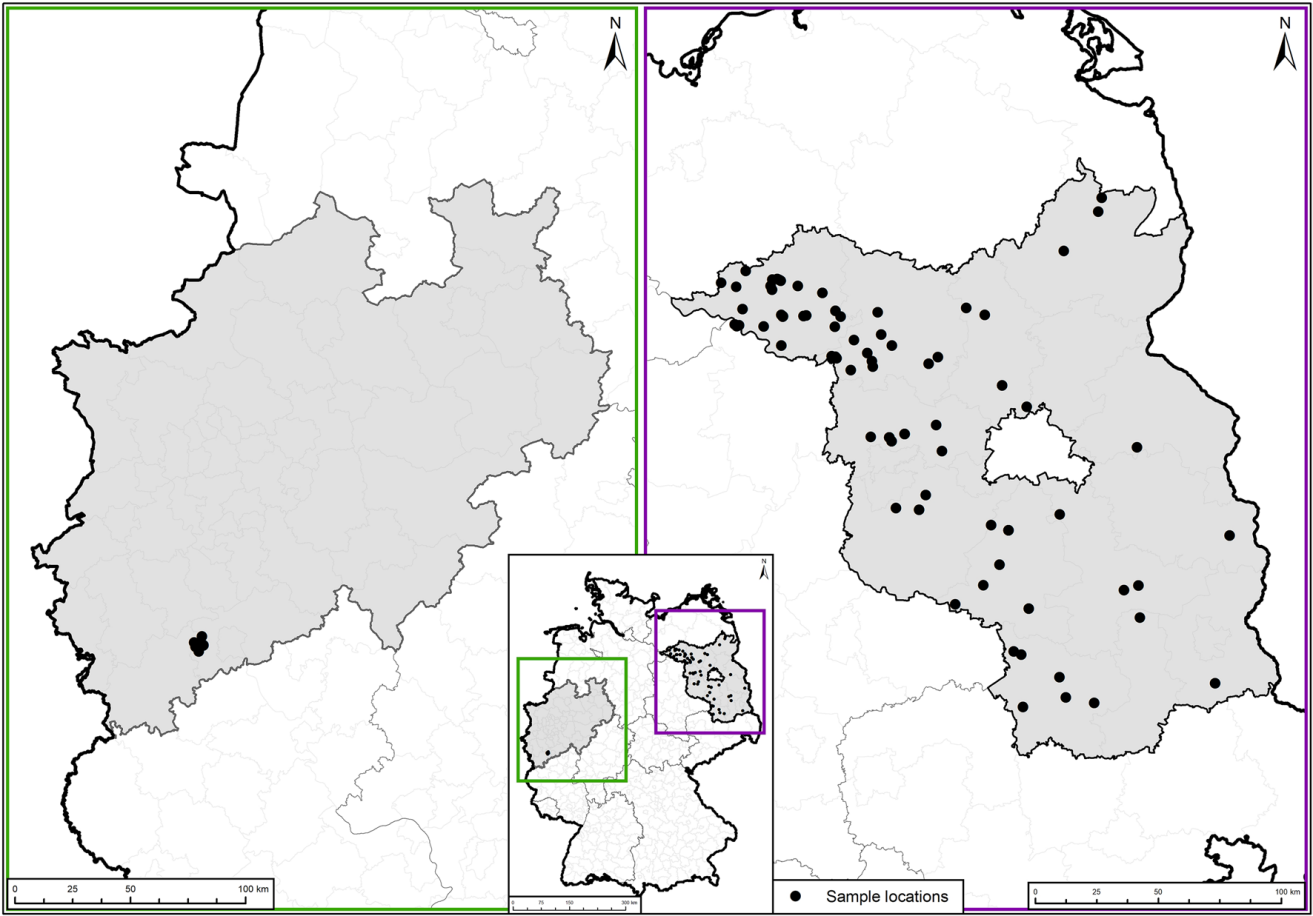
Maps presenting the study areas, which comprised the German federal state of Brandenburg (right panel) and, as a spatial outgroup, a single hunting ground in the Rhein-Sieg-Kreis, North Rhine-Westphalia (left panel). The panel in the center illustrates the locations of the study areas in Germany. Black dots indicate the sampling locations

The study area comprised the German federal state of Brandenburg and, as a spatial outgroup, a single hunting ground in the Rhein-Sieg-Kreis, North Rhine-Westphalia (Fig. 1).

### Selection of *E. multilocularis* specimens

Between 2009 and 2012, a total of 5954 foxes (*Vulpes vulpes*) were examined for *E. multilocularis* after necropsy. Each fox was given a unique identifier (fox ID), which was recorded together with the date and place (geographical coordinates) where the animal was hunted or found, gender (male, female or unknown), and age (juvenile, adult or unknown). Most of the animals were shot by hunters, while a small number were found dead. *Echinococcus multilocularis* was detected in 791 (13.3%) of these foxes using the highly sensitive intestinal scraping technique (IST; [37]). The worm burdens were assessed for these positive foxes (Additional file 1: Figure S1). An intensity of more than five adult *E. multilocularis* was observed in 584 of these foxes (Additional file 1: Figure S1). A total of 91 *E. multilocularis*-positive foxes with more than five parasites were randomly selected and the parasites isolated. Ten *E.* *multilocularis*-positive foxes were randomly chosen from the hunting ground in North Rhine-Westphalia. This resulted in 505 individual *E. multilocularis* specimens that were used for analysis.

Frozen (−20 °C) samples of the mucosa of the large intestine of foxes that had tested positive for *E. multilocularis* by IST were thawed and spread in a petri dish, and five adult *E. multilocularis* were carefully selected using a preparation needle, without damaging the parasite, and transferred into separate microtubes for each adult worm. The microtubes were labeled with the respective fox ID and a unique identifier for the parasite (Em ID).

DNA of five different *E. multilocularis* isolates from Switzerland were used as controls in the EmsB microsatellite fragment length analyses for profiles D, E, G, and H. According to Knapp et al. (2009), profile D corresponds to G05, profile G to G18/G19, and profile H to G28. Profile E has only been described in Switzerland (Fribourg) [17].

### DNA isolation

DNA was isolated from the individual *E. multilocularis* specimens using the High Pure PCR Template Preparation Kit (Roche, Mannheim, Germany) according to the manufacturer’s instructions. A positive processing control prepared from 10 adult *E. multilocularis* specimens derived from a single fox with a large worm burden (Fu/2002/324) and a negative processing control consisting of 25 µl distilled water were included in each DNA isolation. The amount of DNA in each sample was quantified by spectral photometry in a NanoPhotometer P330 with lid 10 (IMPLEN, Munich, Germany) and by applying the Lambert–Beer equation.

### Polymerase chain reaction (PCR)

All reactions (for mitochondrial and EmsB targets) were performed in a volume of 25 µl using the Platinum^®^ Taq DNA Polymerase Kit (Invitrogen, Thermo Fisher Scientific Germany, Braunschweig, Germany) and dNTPs obtained from STRATEC Molecular GmbH, Berlin, Germany, 10 ng template DNA, and the primers listed in Table 1. Primers were purchased from Eurofins Genomics, Ebersberg, or Sigma-Aldrich, Hamburg, Germany. The PCR was conducted in a FlexCycler thermocycler (Analytik Jena AG, Jena, Germany) under the following conditions: initial denaturation for 5 min at 94 °C, 40 cycles with 1 min denaturation at 94 °C, 1 min annealing at 55 °C, 1 min elongation at 72 °C, followed by a terminal elongation of 5 min at 72 °C.

**Table 1:** Primers

Designation	Nucleotide sequence	Target	Reference
co1 for	TTG AAT TTG CCA CGT TTG AAT GC	*Cox1*	Xiao et al. [56]
co1rev	GAA CCT AAC GAC ATA ACA TAA TGA	*Cox1*	Xiao et al. [56]
nd1 for (JB11)	AGA TTC GTA AGG GGC CTA ATA	*Nad1*	Bowles und McManus et al. [8]
nd1 rev (JB12)	ACC ACT AAC TAA TTC ACT TTC	*Nad1*	Bowles und McManus et al. [8]
atp1st for	GTT GTC CGT TAA ATT TCT TTT AGC	*ATP6*	
atp1st rev	GGA ATA ATT GCT AAC CTA CAC AAC	*ATP6*	
EmsB A for	GTG TGG ATG AGT GTG CCA TC	EmsB	Bart et al. [14]
EmsB C rev	[6FAM]CCA CCT TCC CTA CTG CAA TC	EmsB	Bart et al. [14]

Electrophoresis in 1.5% agarose gel was used to check the length and purity of amplicons and to separate DNA fragments of different sizes.

### DNA sequencing

Amplicons were purified using the QIAquick^®^ PCR Purification Kit (QIAGEN, Hilden, Germany) according to the manufacturer’s instructions. For sequencing, they were sent to GATC Biotech AG (Cologne, Germany) using the LightRun Sanger sequencing service, or sequencing was performed with the reverse primers for *Cox1*, *Nad1,* and *ATP6* listed in Table 1 using the BigDye^®^ Terminator v1.1 Cycle Sequencing Kit (Applied Biosystems, Braunschweig, Germany). PCR for sequencing preparation was done in a FlexCycler thermocycler (Analytik Jena AG, Jena, Germany) using the BigDye^®^ Terminator v1.1 Cycle Sequencing Kit. Each sequencing run was conducted under the following cycling conditions: denaturation for 10 s at 96 °C, primer annealing for 5 s at 50 °C, and elongation for 4 min at 60 °C. The samples were then purified using SigmaSpin Post-Reaction Clean-Up Columns (Sigma-Aldrich Biochemie GmbH, Hamburg, Germany) according to the manufacturer’s instructions. Ten microliters of Hi-Di™ formamide was added to approximately 10 µl purified reaction product and analyzed in an ABI 3130 Genetic Analyzer (Applied Biosystems, Thermo Fisher Scientific, Braunschweig, Germany).

Sequences were handled and analyzed using Geneious^®^ version 8.3.1 software (http://www.geneious.com, Biomatters, Auckland, New Zealand) and compared with those of other helminths deposited in the NCBI database (http://www.ncbi.nlm.nih.gov/nucleotide/) using BLAST^®^ (http://blast.ncbi.nlm.nih.gov/Blast.cgi).

### EmsB analyses

EmsB analyses were performed essentially as previously described [16, 17]. In brief, PCR was conducted as described above with the primers EmsB A for and EmsB C rev, which was labeled with 6-carboxyfluorescein (6-FAM; Table 1). Amplicons were shipped to SMB Services in Molecular Biology GmbH, Berlin, Germany, for microsatellite length determination by capillary electrophoresis using the ABI Prism 3100 Genetic Analyzer (Life Technologies, Foster City, CA, USA). EmsB microsatellite profiles were visually determined by comparison with standards using GeneScan 500 ROX (Thermo Fisher Scientific, Waltham, MA, USA). Data were analyzed using GeneMapper^®^ Software 5 (Life Technologies GmbH, Darmstadt, Germany).

### Phylogenetic analyses

Phylogenetic studies were performed using Geneious, MEGA Version 6 (http://www.megasoftware.net/; [38], and R (R Foundation for Statistical Computing, Vienna, Austria, https://www.R-project.org).

For the phylogenetic analysis of the nucleotide sequences, the Hasegawa-Kishino-Yano (HKY) model [39] was applied, using the discrete gamma distribution (+G) and allowing invariant positions (+I) [40].

### Statistical analyses and graphical visualization

Statistical analyses were carried out using Excel (Microsoft Corporation, Redmond, WA, USA), R software version 3.5.3 (R Foundation for Statistical Computing, Vienna, Austria, https://www.R-project.org), and SaTScan v9.3.1 using the Bernoulli model with 999 Monte Carlo replications and allowing a maximum spatial cluster size of 50% of population at risk (Software for the spatial and space–time scan statistics, http://www.satscan.org/ [41]). Fisher's exact test was performed in R with the function “fisher.test” implemented in the R package “stats.” *P*-values < 0.05 were regarded as statistically significant. Bonferroni corrections [42] were performed in multiple testing settings.

For the grouping of the visually determined EmsB microsatellite profiles, the supervised Kohonen network (KN), also called Kohonen maps (self-organizing Kohonen network/self-organizing Kohonen maps [SOM]; [43–45]), was used. For this purpose, the standardized numerical EmsB profiles as described elsewhere [20, 27, 46] were used as input data (x-map). The expected number of groups was set in the program (output data; y-map). The classification was then performed using the “xyf” function of the R package “kohonen.” The topology of the grid was hexagonal, and 10,000 iterations were performed.

To validate the EmsB profile groups obtained by supervised KN, we also performed an unsupervised KN. The grid size for the map space was set to four samples per node, resulting in a grid size of 12 × 12 [44, 45]. The groups/clusters obtained in the unsupervised KN analysis were classified by a combination of the average silhouette method [47], r-function “fviz_nbclust” from the R package “factoextra,” and 30 indices for determining the best clustering scheme from the different results obtained by varying all possible combinations of the numbers of clusters, distance measures, and clustering methods (r-function “NbClust” from the R package “NbClust”) [48, 49]. Both methods proposed a cluster size of four groups as the optimum.

As a second method for checking the EmsB profiles, Sammon’s nonlinear mapping with *k*-means clustering was used [50]. For this purpose, the standardized numerical EmsB profiles determined in this work were used as the data basis and the distances between them were calculated. This calculation was performed with the function “dist” of the R package “stats.” Based on the distances, data were analyzed using the R function “sammon” of the R package “MASS.” The *k*-means cluster analysis was performed using “kmeans” of the R package “stats” [51]. The analysis was performed under the assumption that there were three clusters.

To validate the visual assignment of EmsB profiles and to make the results comparable to previously published data [52], hierarchical clustering analysis (Euclidian distance, average link clustering method) was carried out using in the R package “pvclust.” To improve the reading of the dendrogram, it was modified using the “hang.dendrogram” command from the R package “dendexted” setting the parameter “hang = 0.1.” This parameter changes the fraction of the plot height, in which labels hang below the rest of the plot. A negative value causes the labels to hang down from 0.

Approximate unbiased *P*-values (italic numbers on nodes, in percent) were calculated with a multiscale bootstrap (*B* = 1000). The ArcGIS 10.0 program (Esri, Redlands, CA, USA) was used to visualize geographical data.

## Results

### Mitochondrial markers

DNA sequences/fragments for the *Cox1* derived gene marker (785 bp) could be obtained in 472 of the 505 specimens (93.5%). For 85 of 101 foxes (84.2%), the *Cox1* marker was sequenced for all five worm isolates obtained from each fox. Four different single-nucleotide polymorphisms (SNP) were detected in the sequence alignment of the 472 samples (Additional file 1: Figure S2) for an average of 785 bp sequenced of the 1608 bp of the complete *Cox1* gene (48.8%).

### *Cox1*

Phylogenetic analysis of the *Cox1* gene part sequences, including all the *Echinococcus* species, assigned all samples examined here to the species *E. multilocularis* (Additional file 1: Figure S3). These samples form a separate clade within the monophylum *E. multilocularis*. The *Cox1* data also confirm the monophyletic group that includes *E. ortleppi* (G5) and the genotypes G8, G7, and G6 belonging to the *E. canadensis* cluster, with high bootstrap values. *E. oligarthra* is a sister group of this clade in this analysis, but with low bootstrap support. *Echinococcus equinus* (G4) and *E. granulosus *sensu stricto (s.s.) (G1 and G3) also form a clade together. Both *E. shiquicus* and *E. vogeli* are assigned to independent branches.

One SNP in a *Cox1* gene fragment was detected in a single specimen obtained from fox Fu/2009/1607 and in all five worm specimens from fox Fu/2011/1869 at position 9528 (G to T) as compared to the reference sequence AB018440 (obtained from an alveolar lesion isolated from a naturally infected vole [*Clethrionomys rufocanus*] in Hokkaido, Japan) (https://www.ncbi.nlm.nih.gov/nucleotide/) [53]. One *E. multilocularis* specimen isolated from fox Fu/2011/420 showed a different SNP at nucleotide position 10146 (C to T). The remaining four parasite isolates did not differ from the other isolates from Brandenburg and North Rhine-Westphalia with regard to the *Cox1* marker sequence. At nucleotide position 9625, a further SNP was detected in two parasite isolates of fox Fu/2012/1527 (C to T). Unfortunately, not all five worm isolates obtained from this fox could be sequenced. One out of five *E. multilocularis* specimens isolated from fox Fu/2011/564, showed a SNP at position 9638 (A to G).

Three of the four SNPs resulted in an amino acid exchange. Leucine was replaced by phenylalanine, glycine by cysteine, and alanine by valine.

### *Nad1*

The sequence of the *Nad1* gene fragment was determined for 470 specimens (93.1%). This marker was sequenced in all five worm isolates from 84 foxes (83.2%). Only two different SNPs were detected in the *Nad1* gene (Additional file 1: Figure S4) in an average of 379 bp sequenced of 894 bp of the complete *Nad1* gene (42.4%).

Based on the phylogenetic analysis performed with the *Nad1* sequences, the isolates of the present study could also be assigned to the species *E. multilocularis* (Additional file 1: Figure S5). Our samples again form a separate clade within the monophylum *E. multilocularis*. Here, the *E. multilocularis* clade is a sister group of *E. shiquicus*. All clades were confirmed with high bootstrap values. The same is true for the monophyletic group formed by *E. ortleppi* (G5) and the G8, G7, and G6 genotypes from the *E. canadensis* cluster. *Echinococcus granulosus *s.s. (G1 and 3) and *E. equinus* (G4) were each assigned to an independent branch between these clades. The bootstrap ratios are lower than in the clades described above. Both *E. oligarthra* and *E. vogeli* form independent branches in this analysis.

Three of the five worm specimens isolated from fox Fu/2009/2374 displayed a SNP in *Nad1* at position 7911 (G to A). All five parasite specimens obtained from fox Fu/2009/1860 showed a SNP at nucleotide position 8030 (A to G). One of the two SNPs resulted in an amino acid exchange (glycine to serine).

### *ATP6*

Sequences for *ATP6* could be determined in 479 samples (94.9%). The marker was sequenced in all five worm isolates recovered from 88 foxes (87.1%). Four different SNPs were detected in the sequence alignment of the *ATP6* gene (Additional file 1: Figure S6) in an average of 516 bp, i.e. the complete *ATP6* gene (100%).

Phylogenetic analysis revealed that all *ATP6* gene sequences determined in this study could be assigned to the species *E. multilocularis* (Additional file 1: Figure S7). Here, *E. vogeli* forms a clade with *E. multilocularis*, but with little bootstrap support. The monophyletic group, which includes *E. ortleppi* and the genotypes G8, G7, and G6 from the *E. canadensis* cluster, could be confirmed with high bootstrap values. *Echinococcus oligarthra* is a sister group of this clade, but with low bootstrap support. *Echinococcus equinus* and *E. granulosus *s.s. together also form a clade. *Echinococcus shiquicus* was assigned to an independent branch.

In three foxes (Fu/2011/1533, Fu/2011/1551, and Fu/2009/1042), a SNP was detected at the same nucleotide position (6147) in at least one of the five worm sequences (C to T). Four of five parasite sequences of fox Fu/2011/1551 showed a change at this nucleotide position. In the cases of foxes Fu/2011/1533 and Fu/2009/1042, this SNP was only detected in one of the five parasite specimens recovered from these animals. In all five *E. multilocularis* specimens recovered from fox Fu/2012/1590, a SNP was detected at nucleotide position 5934 (T to C), which was not found in any parasite specimen of any other fox. At position 6247, the *ATP6* sequences of all five parasite isolates obtained from fox Fu/2009/1828 differed from the reference sequence and from all other sequences (C to T). The *ATP6* sequences of all five worm specimens recovered from fox Fu/2009/1860 exhibited a SNP at position 6375 (C to T).

Three of the four SNPs resulted in amino acid changes (alanine to valine, serine to proline, and histidine to tyrosine).

To increase the robustness of previous phylogenetic analyses, the sequence data were concatenated and re-analyzed for a total of 3189 aligned bp. All concatenated sequences determined in this study could be assigned to the species *E. multilocularis* (Additional file 1: Figure S8).

Also with the concatenated dataset, our samples form a separate clade within the monophylum *E. multilocularis*. Here, the *E. multilocularis* clade is a sister group of *E. shiquicus*. Both *E. oligarthra* and *E. vogeli* form independent branches in this analysis. Within the *E. granulosus *s.l. group, *E. ortleppi* and *E. canadensis* genotypes (G6-G8) clustered together. *Echinococcus granulosus* s.s. (G1) and *E. equinus* (G4) are located on separate branches within this group. All clades were confirmed with high bootstrap values (Additional file 1: Figure S8).

In conclusion, infections with mixed genotypes as determined by the *Cox1* marker were detected in four foxes, and another one using the *Nad1* marker in another fox. Three foxes showed multiple infections with *E. multilocularis* genotypes, which differed with regard to the *ATP6* marker.

### EmsB microsatellite analysis and comparison with mitochondrial genotyping

From the total of 505 *E. multilocularis* specimens, EmsB microsatellite profiles could be determined for 490 (97.0%), and EmsB profiles were obtained for all five worm specimens isolated from each fox for 91 out of 101 foxes (90.1%). For 15 (3.0%) specimens, definitive visual determination of the profile was not possible.

We detected four different profiles (D, E, G, and H) and some parasites that could not be unambiguously assigned to an existing profile (designated as K), but were further analyzed as described in the next section. Profile E could only be detected in a single *E. multilocularis* isolate from North Rhine-Westphalia. In 80 of 101 foxes (79.2%), genotyping information for the mitochondrial markers (*Cox1*, *Nad1,* and *ATP6*) and the EmsB profiles could be determined for all five worm isolates obtained from each fox. Profile D was found in 194 (38.4%), G in 257 (50.9%), H in 38 (7.5%), and E in one (0.2%) of the 505 worm specimens. The differences in the proportions of the profiles were statistically significant (Fisher’s exact test; *P*-value = 0.0041).

In 14 foxes (10 from Brandenburg and four from North Rhine-Westphalia) the EmsB profile of at least one *E. multilocularis* specimen differed from the profiles of the remaining four specimens from the respective foxes.

For the foxes Fu/2011/564, Fu/2009/1607, Fu/2011/420, and Fu/2012/1527, in which one worm isolate differed from the remaining four worm isolates by a SNP in the *Cox1* gene, it was shown that the EmsB profile of the respective specimen also differed from the other four specimens obtained from the same fox. Thus, all four *E. multilocularis* specimens from fox Fu/2011/564 belonged to the EmsB profile H, and the specimen Fu/2011/564-5 had the profile D. The profile G was found four times in fox Fu/2009/1607 and profile H once in Fu/2009/1607-3. The SNP in the *Cox1* gene of the worm isolate 3 was also detected in all five parasite specimens isolated from fox Fu/2011/1869. All isolates from this fox belonged to the EmsB profile H. In fox Fu/2011/420, profile D was found in four specimens and profile H in one parasite. In fox Fu/2012/1527, profile D was found four times and profile G once. No agreement was found in EmsB profile heterogeneity with the SNPs in the remaining two mitochondrial markers *Nad1* and *ATP6*.

### Validation of the visual determination of EmsB profiles

To evaluate the reliability of the visual determination of EmsB profiles and to determine their distinctiveness, supervised self-organizing KN analysis was performed and the results displayed in SOM. The standardized numerical EmsB profiles found in this study were used as the data basis. Due to a change in the standard for capillary gel electrophoresis, 38 samples had to be removed from the data set, so that 427 samples remained for KN analysis. Profiles D and E were combined into one group, because only one of the 490 samples for which an EmsB profile could be determined was assigned to profile E. After the number of expected groups (equivalent to the number of expected EmsB profiles) had been set, classification by the KN was carried out using the “xyf” function in R package “kohonen.” In the graphical visualization of the results, circles represent the groups of profiles. Circles with the same color belong to the same group. The visually determined profiles are shown in different colored circle sectors and were assigned to the groups using the “xyf” function.

**Fig. 2:**
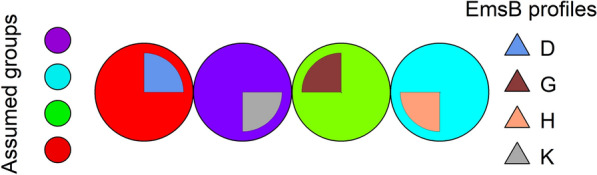
Supervised Kohonen self-organizing network analysis with the assumption of four groups for the EmsB profile data. The results of the grouping of the EmsB profiles by the Kohonen self-organizing network algorithm are shown. The four assumed groups are represented by the colors red, green, purple, and light blue. EmsB profiles D (blue), G (brown), and H (salmon) are indicated in the circle sectors. Isolates that could not be visually assigned to any EmsB profile (K) are represented by the gray circle sector

The analysis revealed four groups predicted by the KN according to the EmsB profiles, which are identified by four different colors (Fig. 2). Each EmsB profile corresponded perfectly to the assumed group. The samples that could not be visually assigned to any profile formed a separate group in this analysis (profile K).

**Fig. 3:**
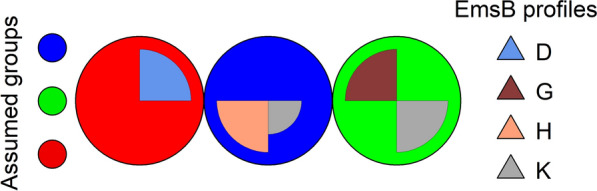
Supervised Kohonen self-organizing network analysis with the assumption of three groups for the EmsB profile data. The results of the grouping of the EmsB profiles by the Kohonen self-organizing network algorithm are shown. The three assumed groups are represented by the colors red, green, and blue. EmsB profiles D (blue), G (brown), and H (salmon) are indicated in the circle sectors. Isolates that could not be visually assigned to any EmsB profile (K) are represented by the gray circle sector

To examine whether the samples that could not be visually assigned to any profile could be grouped with any of the established profiles D, G, or H, the existence of only three groups was assumed and the analysis repeated. Under these conditions, the visually determined profile G and the samples that could not be clearly assigned to any profile (K) formed one group (Fig. 3). Furthermore, a small part of the samples that could not be allocated to any profile (K) clustered with profile H. None of the samples that could not be visually attributed to any profile was allocated to profile D.

**Fig. 4:**
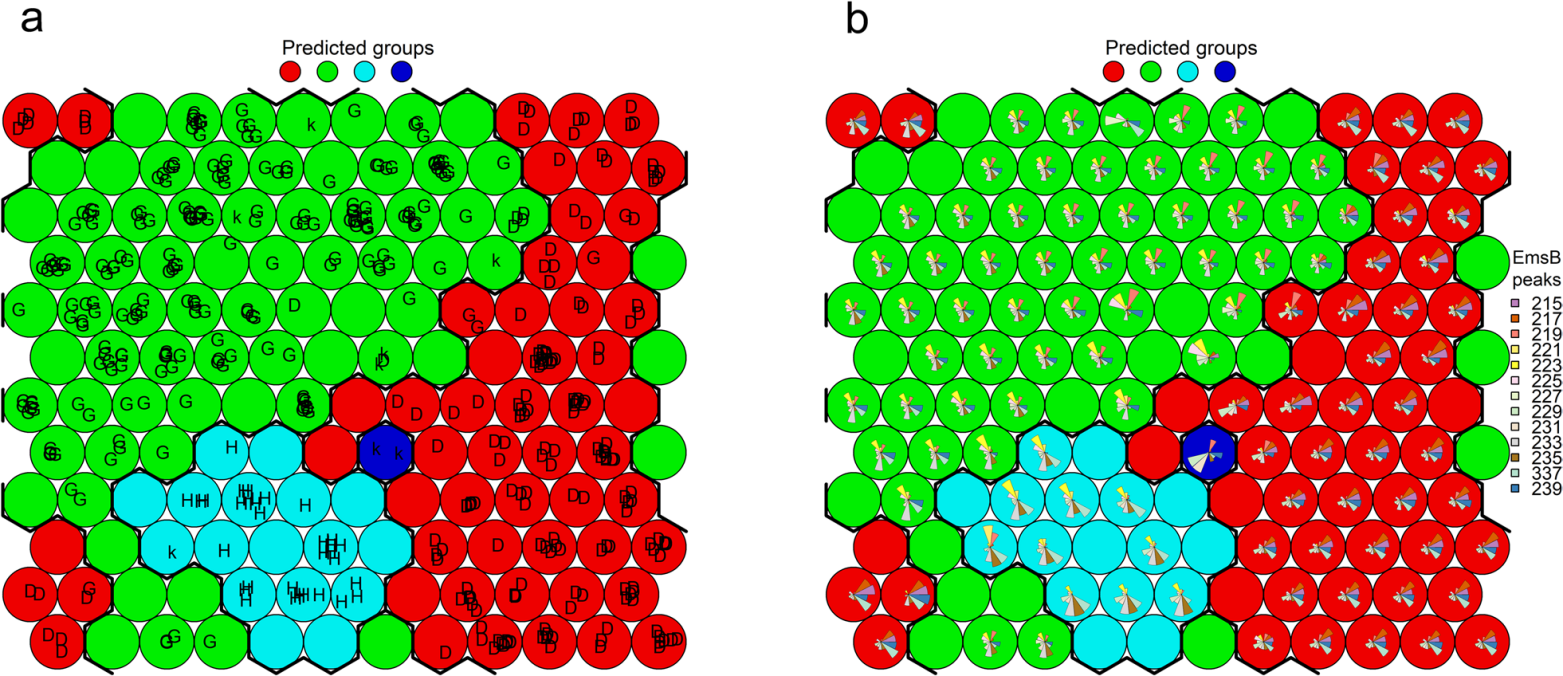
Unsupervised Kohonen self-organizing network analysis of EmsB profile data. The four assumed/predicted groups are represented by the colors red (EmsB profile D), green (EmsB profile G), dark blue (EmsB profile K), and light blue (EmsB profile H) in both panels (**a** and **b**). Panel **a** shows the locations of the samples within the grid nodes annotated according to the EmsB typing. Panel **b** presents the EmsB profiles, according to which the samples were classified in respective grid nodes. Each segment within the node-specific segment pattern represents the value of the EmsB peak

Unsupervised KSN analysis mapped the EmsB data into four groups/clusters, confirming the correctness of the visually classified EmsB profiles (D (red nodes), G (green nodes), H (light blue nodes), and unknown profiles designated as “K” (dark blue node) (Fig. 4a, b).

**Fig. 5:**
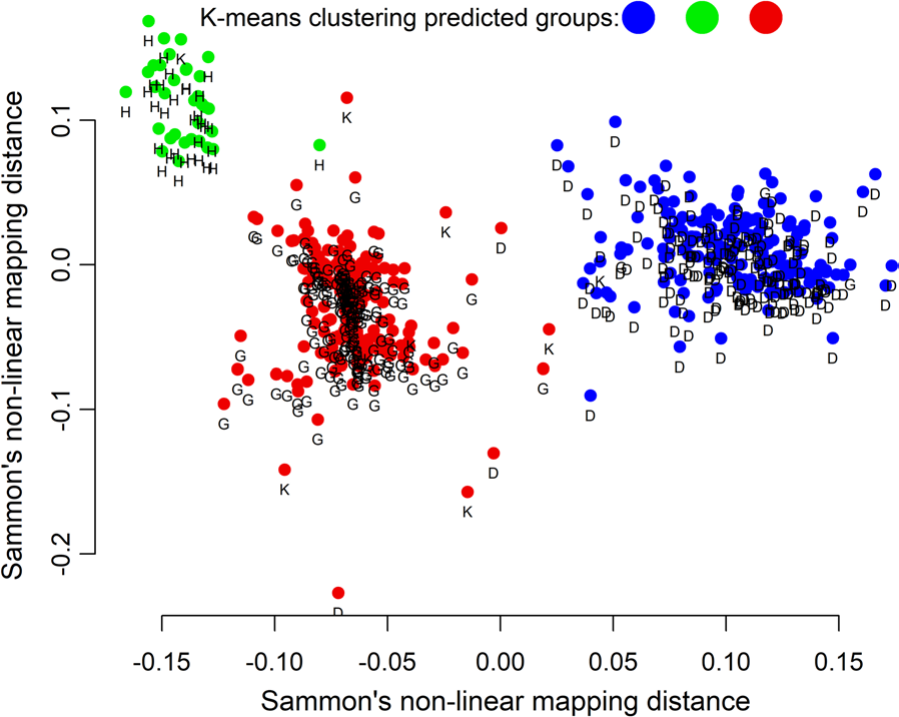
Grouping of EmsB profiles by Sammon’s nonlinear mapping with *k*-means clustering**.** The colors red, green, and blue represent the three clusters. The positions in the graph represent the results of Sammon's nonlinear mapping analysis, with dots labeled with the corresponding EmsB profiles D, G, and H. Samples that could not be visually assigned to any profile are marked by K

The validity of the visual profile determination was further examined by Sammon’s nonlinear mapping with *k*-means clustering. When the existence of three clusters was assumed, the resulting profile groups corresponded to the visually determined profiles (Fig. 5). These groups were spatially separated from each other. The samples which could not be visually assigned to any profile scattered around the cloud representing samples with profile G.

**Fig. 6:**
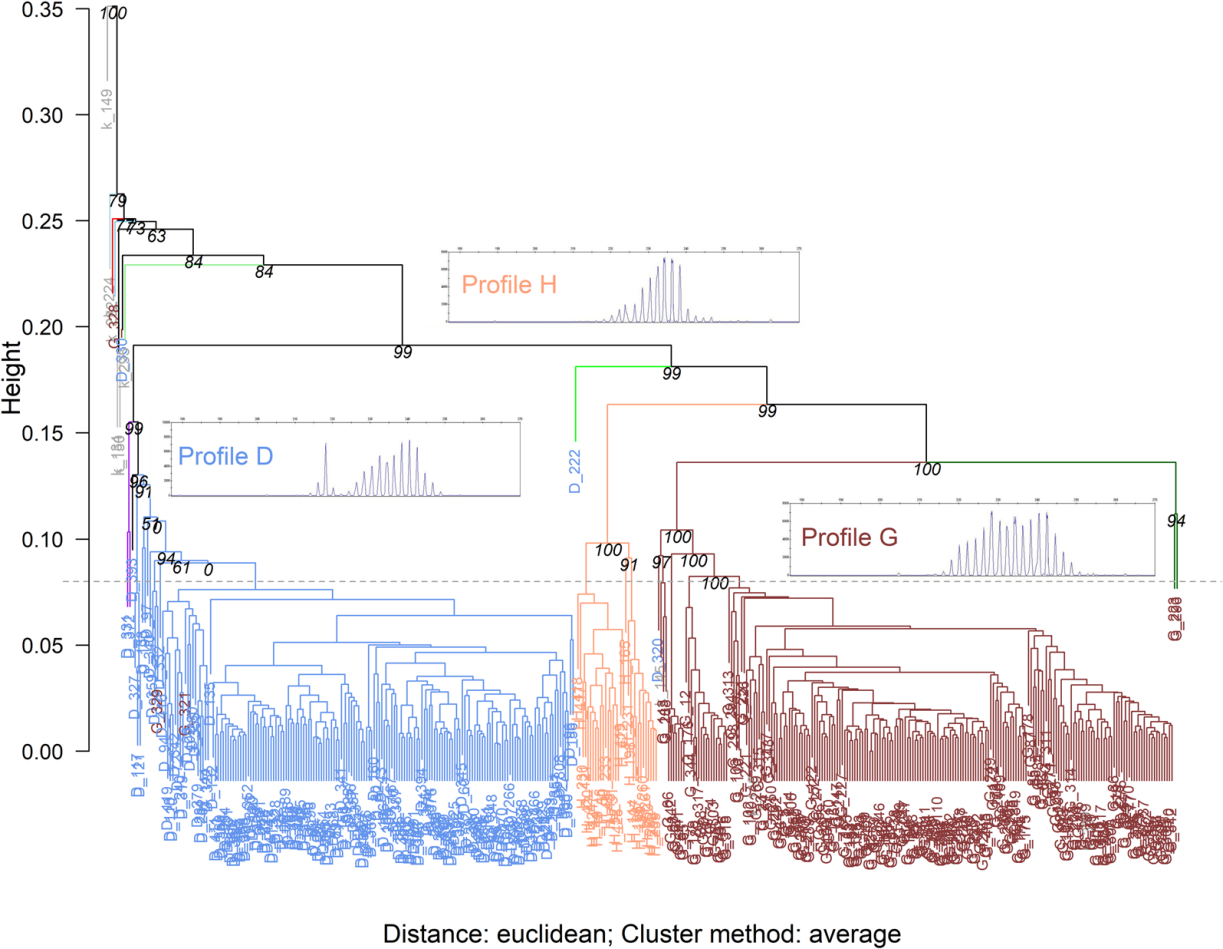
Cluster dendrogram for EmsB profiles of *Echinococcus* *multilocularis*. A dendrogram with *p*-/bootstrapping values shows the clusters of EmsB genotyping data for *E. multilocularis* isolates from Brandenburg and North Rhine-Westphalia, Germany. The colors of the dendrogram branches indicate the clusters. The labels annotating the branches are the IDs of the genotyped isolates and their color represent the genotyping results: blue represent *E. multilocularis* isolates of the EmsB microsatellite profile D; Indian red shows *E. multilocularis* isolates of profile G; light salmon illustrates profile H. In the dendrogram area corresponding to the respective clusters, a typical electropherogram of the EmsB loci (209–241 bp) obtained using the ABI Prism 3100 automatic sequencer is shown and annotated with the respective profile name. The gray horizontal dashed line indicates the threshold in genetic distance at 0.08

Hierarchical clustering analysis also classified the EmsB genotyping data of the *E. multilocularis* isolates into four groups when we applied a threshold of 0.08 for the genetic distance [18] (Fig. 6). The profiles D, G, and H were clustered in three separate clusters, with the fourth cluster consisting mainly of samples representing patterns that could not be assigned to any known EmsB profile and single isolates representing EmsB profiles D or G.

These results confirm in multiple ways the validity of the visual determination of profiles. It seems likely that samples that could not be attributed to any established profile belong to at least one separate profile.

### Spatial distribution of genotypes

Due to the limited number of SNPs in the sequences of the mitochondrial markers *Cox1*, *Nad1,* and *ATP6*, it was not possible to draw any conclusions regarding the spatial distribution of *E. multilocularis* on the level of the federal state of Brandenburg or in the study area in North Rhine-Westphalia (Additional file 1: Figure S9).

**Fig. 7:**
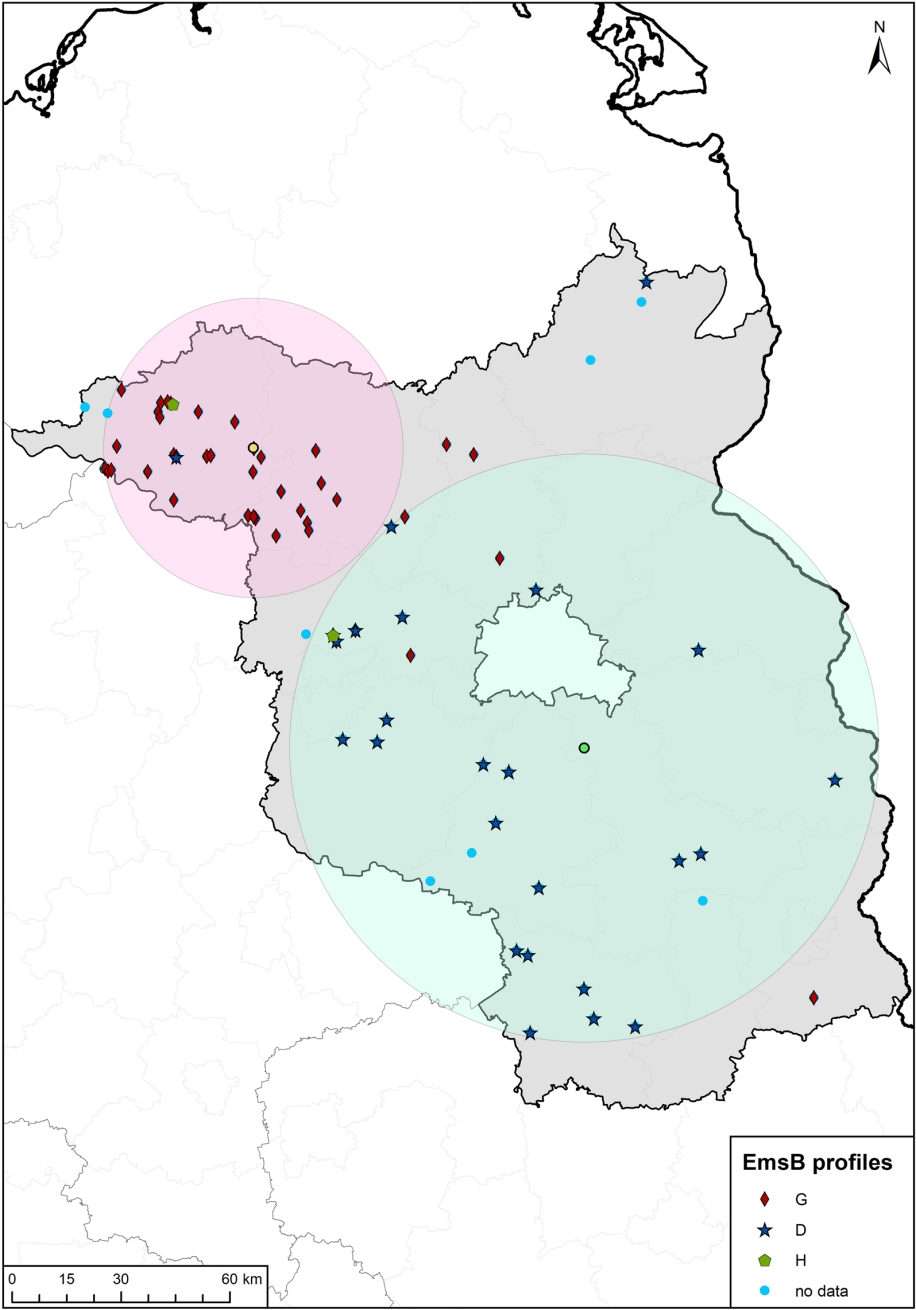
Map of *Echinococcus multilocularis*-positive foxes and respective EmsB profiles of worm isolates. The light purple and light green circles represent spatial clusters confirmed by SaTScan analysis (center of the circle represented by green and yellow dots, respectively). Profiles G (red diamonds), D (blue stars), and H (green pentagons) are indicated. Parasite isolates that could not be clearly assigned to any EmsB profile are marked by blue dots

When the spatial coordinates of *E. multilocularis-*infected foxes (*n* = 90) with identical EmsB profiles for all sampled parasite specimens were plotted on a map, it became evident that profile G was predominantly detected in northwestern Brandenburg in the districts of Prignitz and Ostprignitz-Ruppin (Fig. 7). In contrast, profile D was predominantly found in central and southern Brandenburg. Profile H was found in three of the foxes, two of which came from Brandenburg, and the third one from North Rhine-Westphalia. Profile D was found in two foxes and profile G in a single fox in North Rhine-Westphalia.

Spatial analysis by searching for high rate clusters with a Bernoulli model using SaTScan revealed two clusters (Fig. 7). Cluster 1 is located in northwestern Brandenburg and comprises foxes with the *E. multilocularis* specimens of profile G. Cluster 2 is formed by parasites of the profile D. The geographical center of this profile is south of Berlin in the middle of Brandenburg and extends to the central and southern parts of the federal state. No separate cluster was identified for profile H and the foxes (*n* = 2) assigned to it, nor could this profile be included in one of the two confirmed clusters.

## Discussion

Genotyping of *E.* *multilocularis* may be useful for studying the epidemiology, in particular the spread and distribution, of the parasite in different host populations, and can also significantly contribute to understanding the phylogeny of this cestode. In the present study, we investigated the genotype diversity of *E. multilocularis* in the red fox in two selected study regions in Germany [1, 31, 32]. We used conventional and well-defined markers to individually genotype different specimens of *E. multilocularis* isolated from distinct single hosts, allowing us to determine whether the population of the parasite in an individual host is genetically homogeneous.

Mixed infections of individual foxes with *E. multilocularis* parasites of different genotypes were detected in Brandenburg and North Rhine-Westphalia by means of EmsB microsatellite analysis and with mitochondrial markers. Knapp et al. [17, 18] and Nakao et al. [54] also found such mixed infections. It is interesting that four of ten foxes from North Rhine-Westphalia were infected with parasites of more than one EmsB profile. In Brandenburg, only 10 of 91 selected foxes had mixed infections. In comparison, Knapp et al. [18] found mixed infections in 25 foxes (52%) from France (an area of 900 km^2^ in the Département Ardennes) [18]. In a further study by Knapp and colleagues, mixed infections were found in 44 (35%) of 125 foxes from several European countries [17].

For typing, DNA samples were extracted using individual whole worms. Although deemed unlikely, it seems possible that some of these worms might have harbored eggs fertilized with sperm from *E. multilocularis* worms representing other genotypes, potentially resulting in mixed genotypes. In such cases, the possibility cannot be excluded that the genotyping result does reflect the single-worm genetics, but rather a genetic cross between two worms due to cross-fertilization.

Attempts to genotype parasites of the genus *Echinococcus* and to characterize subpopulations started with mitochondrial markers [7, 8]. While the genetic diversity of *E. granulosus *s.l. became obvious, that of *E. multilocularis* appeared much less pronounced, when similar markers were used. However, haplotypes with a geographically distinct distribution clustering in European, Asian, and North American clades could be demonstrated [7–9, 11, 55]. We included the mitochondrial markers from *Cox1*, *Nad1,* and *ATP6* gene regions in our studies, as these markers were used in former studies [8, 56] and therefore comparison data are available [57, 58]. Genetic variability within *E. multilocularis* has also been extensively studied using the microsatellite marker EmsB [16]. We therefore combined these markers in our analysis to characterize the *E. multilocularis* population genetically in the federal state of Brandenburg and to make the results comparable to other studies.

The genotyping results obtained by sequencing target regions of mitochondrial markers *Cox1*, *Nad1,* and *ATP6* and the respective phylogenetic analyses show that the *E. multilocularis* specimens we examined belong to the same clade, as expected. However, these markers do not allow a more detailed geographical analysis. The EmsB marker was much more promising in this respect.

To ensure the comparability of the EmsB microsatellite analysis with published data, a visual evaluation of the obtained EmsB profiles was performed using the profile descriptions by Knapp et al. [16], who applied five main profiles (D–H) for European *E. multilocularis* isolates. An EmsB profile does not necessarily represent a single genotype. One profile (e.g. D, G, or H) can consist of different genotypes, as described in the original papers [16, 17] and also clearly evident from Fig. 6. The height of each peak in each profile can vary slightly due to different numbers of repeats in the genome, leading to the respective peak in the microsatellite analysis [14]. Since visual inspection of EmsB profiles is not entirely based on measurable quantitative effects, but has an inherent arbitrary element, we validated the visual determination of the profiles by a KN analysis [43] and Sammon’s nonlinear mapping with *k*-means clustering [50, 51].

In the Kohonen self-organizing network analysis, a self-organizing network is based on a data set, on which the network is trained to recognize patterns [43–45, 59]. The individual data are then assigned to the respective groups. In our study, we trained the system with the numerical data of the determined profiles from Germany and reference samples from Switzerland. The aim of this study was to investigate the genetic diversity of *E. multilocularis* in the federal state of Brandenburg in foxes as definitive hosts, where we found a total of three known and one unknown EmsB profile. Previously published EmsB data from Germany [20], where genotyped isolates originated from human cases of alveolar echinococcosis, showed a significantly higher diversity (11 EmsB profiles) compared to the results of this study. Because of the different study setups, the previously published data were not included in the analysis in this study.

The result of the analysis confirmed the existence of four groups of isolates in the study area, three of which could be attributed to existing EmsB profiles, whereas one comprised isolates that could not be visually assigned to any known EmsB profile. This finding was also confirmed by Sammon’s nonlinear mapping with *k*-means clustering. This method was originally developed to identify, compare, and group spectra resulting from matrix-assisted laser desorption ionization-time of flight mass spectrometry (MALDI-TOF MS) [51].

The analyses resulted in three clusters, to which the samples with the profiles H, G, and D were assigned. On the maps produced by the algorithm, samples that visually could not be assigned to any profile clustered around profile G. This result is consistent with that of the KN analysis under the assumption of the existence of three groups. However, these samples (marked “K”) could also be clearly distinguished from profile G when the EmsB profiles were visually examined. As neither visual inspection nor KN analysis nor Sammon’s nonlinear mapping with *k*-means clustering could assign samples unambiguously to a profile, it remains open whether they represent a separate profile or could not be attributed to any known cluster, perhaps due to poor sample quality.

Four profiles were identified in the samples from Brandenburg and North Rhine-Westphalia. The profiles D and G were found most frequently. Profile E could only be detected in a single *E. multilocularis* isolate from North Rhine-Westphalia. Profile H was identified in 38 worm specimens.

In the present study, the EmsB microsatellite profile was determined for 490 of a total of 505 *E. multilocularis* specimens. In comparison, 81 worm isolates were examined in a study with samples from Hungary [60], where profile H was most common (55.5%). In our study, profile H was underrepresented. Its low proportion differed statistically significantly from those of the specimens attributed to profiles G and D. Profile G was found with a proportion of 50.9% and thus represented the most frequently occurring profile in the study area.

Spatial analysis of the EmsB profiles in Brandenburg revealed two geographically distinguishable clusters with different EmsB profiles. One of the two groups, characterized by profile G, is located in an endemic area in the districts of Prignitz and Ostprignitz-Ruppin and was already discovered in the 1990s [32]. The second cluster, formed by *E. multilocularis* specimens of the profile D, was located in central and southern Brandenburg. It is tempting to speculate that this genotype migrated into Brandenburg from neighboring areas in Saxony-Anhalt or from the Czech Republic. According to Knapp et al. (2009), the profile D is also described as a profile “G05” and is well represented in Germany and the Czech Republic. This view is supported by findings of Denzin et al. [61], who showed a shift in the geographical center of *E. multilocularis* by 3.4 km per year in a north-northeast direction. To prove the suggested link between *E. multilocularis* in Saxony-Anhalt and Brandenburg, parasite specimens from Saxony-Anhalt need to be genotyped.

The EmsB marker results also support the hypothesis that the study areas in Brandenburg and North Rhine-Westphalia have long been endemic for *E. multilocularis*, as at least three clearly different EmsB profiles were found. The existence of at least two statistically significantly separate clusters in Brandenburg may suggest that different disease dynamics dominate in the regions where the discernable clusters have formed. In Brandenburg, we found no evidence for a core region from which the parasite had spread [55]. The existence of two geographically distinct worm populations in Brandenburg may suggest that long-distance fox migration does not play a major role in the spread of *E. multilocularis*.

It seems essential to collect, genotype, and compare more *E. multilocularis* specimens from different regions to further validate the discriminative power of EmsB and other markers and to perform more detailed spatial and temporal analyses regarding the distribution or spread of the parasite. We therefore fully support the EmsB Website for the *Echinococcus* Typing [46] and will be pleased to submit our genotyping data once an automatic upload function is implemented.

Further markers should be identified, for example by next-generation sequencing (NGS) approaches, to improve the genotyping of *E. multilocularis*. This strategy is promising and may provide high discriminative power in *E. multilocularis* populations and allow precise identification of polymorphic and conserved regions. Such regions might also be used as potential markers for the typing of *E. multilocularis* at different levels, i.e. development of “fingerprinting” assays providing different resolution levels in genotyping.

## Conclusions

Genotyping of *E. multilocularis* specimens from Brandenburg and North Rhine-Westphalia, Germany, showed that individual foxes may harbor different genotypes of the parasite. EmsB microsatellite proved suitable for identifying parasite clusters at the regional level, which may help to understand the distribution and spread of the infection in wildlife in endemic areas.

## Supplementary Information

**Additional file 1: Figure S1**. Total number of foxes naturally infected with *E. multilocularis* tested by intestinal scraping technique (IST). The positive animals were stratified according to the worm burden as recommended by the World Health Organization (WHO). +: 1–5 worms; ++: >5–50 worms; +++: >50–1000 worms; ++++: >1000 worms. **Figure S2**. Alignment of the *Cox1* nucleotide sequences of isolates from Brandenburg and North Rhine-Westphalia and the reference sequence AB018440. The *Cox1* gene is shown (yellow bar). The black bars represent the position of the SNPs in the sequences of the parasite isolates compared to the consensus sequence of all samples investigated in this work. **Figure S3**. Dendrogram derived from the *Cox1* gene data of the consensus sequence of all isolates identified in this work, the sequences with the respective SNPs and the sequences taken from Nakao et al. [62]. Bootstrap values (%) are shown on the branches. **Figure S4**. Alignment of the *Nad1* nucleotide sequences of isolates from Brandenburg and North Rhine-Westphalia and the reference sequence AB018440. The *Nad1* gene is shown (yellow bar). The black bars represent the position of the SNPs in the sequences of the parasite isolates compared to the consensus sequence of all samples investigated in this work. **Figure S5**. Dendrogram derived from the *Nad1* gene data of the consensus sequence of all isolates identified in this work, the sequences with the respective SNPs and the sequences taken from Nakao et al. [62]. Bootstrap values (%) are shown on the branches. **Figure S6**. Alignment of the *ATP6* nucleotide sequences of isolates from Brandenburg and North Rhine-Westphalia and the reference sequence AB018440. The *ATP6* gene is shown (yellow bar). The black bars represent the position of the SNPs in the sequences of the parasite isolates compared to the consensus sequence of all samples investigated in this work. **Figure S7**. Dendrogram derived from the *ATP6* gene data of the consensus sequence of all isolates identified in this work, the sequences with the respective SNPs and the sequences taken from Nakao et al. [62]. Bootstrap values (%) are shown on the branches. **Figure S8**. Dendrogram derived from the concatenated (atp6, cox1, nd1) data of the consensus DNA sequences of all isolates identified in this work, the respective SNPs and the sequences taken from Nakao et al. [62]. Bootstrap values (%) are shown on the branches.

## Data Availability

The datasets used or analyzed during the current study are available from the corresponding author on reasonable request.
