# Supplementary material for: Red foxes harbor two genetically distinct, spatially separated Echinococcus multilocularis clusters in Brandenburg, Germany
Source: Parasit Vectors. 2021 Oct 14;14:535. doi: 10.1186/s13071-021-05038-0 (PMC8518320; doi:10.1186/s13071-021-05038-0)
Supplement: Supplementary file 1 — Additional file 1: Figure S1. Total number of foxes naturally infected with E. multilocularis tested by intestinal scraping technique (IST). The positive animals were stratified according to the worm burden as recommended by the World Health Organization (WHO). +: 1–5 worms; ++: >5–50 worms; +++: >50–1000 worms; ++++: >1000 worms. Figure S2. Alignment of the Cox1 nucleotide sequences of isolates from Brandenburg and North Rhine-Westphalia and the reference sequence AB018440. The Cox1 gene is shown (yellow bar). The black bars represent the position of the SNPs in the sequences of the parasite isolates compared to the consensus sequence of all samples investigated in this work. Figure S3. Dendrogram derived from the Cox1 gene data of the consensus sequence of all isolates identified in this work, the sequences with the respective SNPs and the sequences taken from Nakao et al. [62]. Bootstrap values (%) are shown on the branches. Figure S4. Alignment of the Nad1 nucleotide sequences of isolates from Brandenburg and North Rhine-Westphalia and the reference sequence AB018440. The Nad1 gene is shown (yellow bar). The black bars represent the position of the SNPs in the sequences of the parasite isolates compared to the consensus sequence of all samples investigated in this work. Figure S5. Dendrogram derived from the Nad1 gene data of the consensus sequence of all isolates identified in this work, the sequences with the respective SNPs and the sequences taken from Nakao et al. [62]. Bootstrap values (%) are shown on the branches. Figure S6. Alignment of the ATP6 nucleotide sequences of isolates from Brandenburg and North Rhine-Westphalia and the reference sequence AB018440. The ATP6 gene is shown (yellow bar). The black bars represent the position of the SNPs in the sequences of the parasite isolates compared to the consensus sequence of all samples investigated in this work. Figure S7. Dendrogram derived from the ATP6 gene data of the consensus sequence of all isolates [file 13071_2021_5038_MOESM1_ESM.docx]

**Red foxes harbor two genetically distinct, spatially separated *Echinococcus multilocularis* clusters in Brandenburg, Germany**

Mandy Herzig^1#^, Pavlo Maksimov^1#^, Christoph Staubach^1^, Thomas Romig^2^, Jenny Knapp^3,4^, Bruno Gottstein^5^, Franz J. Conraths^1^*

#Mandy Herzig and Pavlo Maksimov contributed equally to this work.

^1^Friedrich-Loeffler-Institut, Federal Research Institute for Animal Health, Institute of Epidemiology, Südufer 10, 17493 Greifswald-Insel Riems, Germany

^2^Universität Hohenheim, Institut für Biologie, Fachgebiet Parasitologie, Emil-Wolff-Straße 34, 70599 Stuttgart, Germany

^3^UMR CNRS 6249 Laboratoire Chrono-environnement, Université Bourgogne Franche-Comté, 16 Route de Gray, 25030 Besançon, France

^4^Department of Parasitology-Mycology, National Reference Centre for Echinococcoses, University Hospital of Besançon, 25030 Besançon, France

^5^Institute for Infectious Diseases, Faculty of Medicine, University of Berne, 3001 Berne, Switzerland

*Correspondence:

Professor Dr. Franz J. Conraths

Friedrich-Loeffler-Institut

Südufer 10

17493 Greifswald-Insel Riems

Germany

Phone: +49 38351 71522

E-Mail: franz.conraths@fli.de


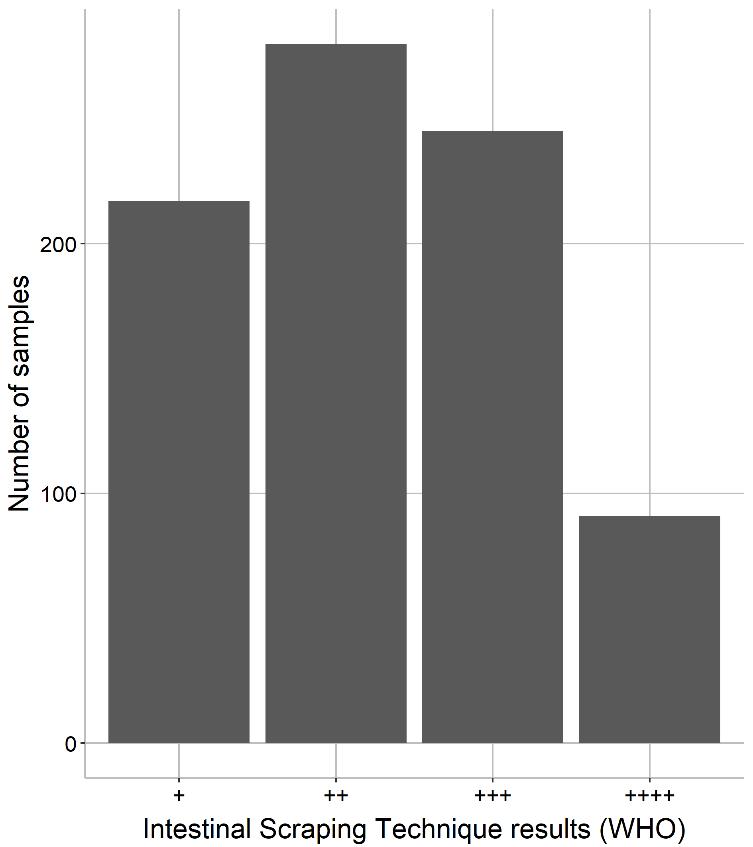


**Additional file 1: Figure S1.** Total number of foxes naturally infected with *E. multilocularis* tested by Intestinal Scraping Technique (IST). The positive animals were stratified according to the worm burden as recommended by the World Health Organization (WHO). +: 1-5 worms; ++: >5-50 worms; +++: >50-1000 worms; ++++: >1000 worms.


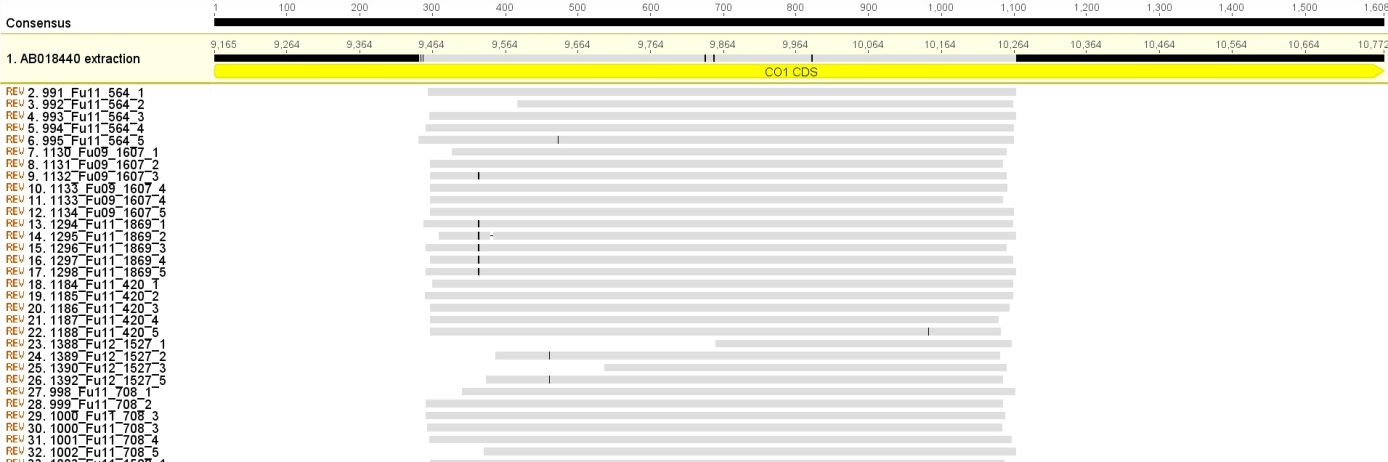


Additional file 1: Figure S2. Alignment of the *Cox1* nucleotide sequences of isolates from Brandenburg and North Rhine-Westphalia and the reference sequence AB018440. The *Cox1* gene is shown (yellow bar). The black bars represent the position of the SNPs in the sequences of the parasite isolates compared to the consensus sequence of all samples investigated in this work.


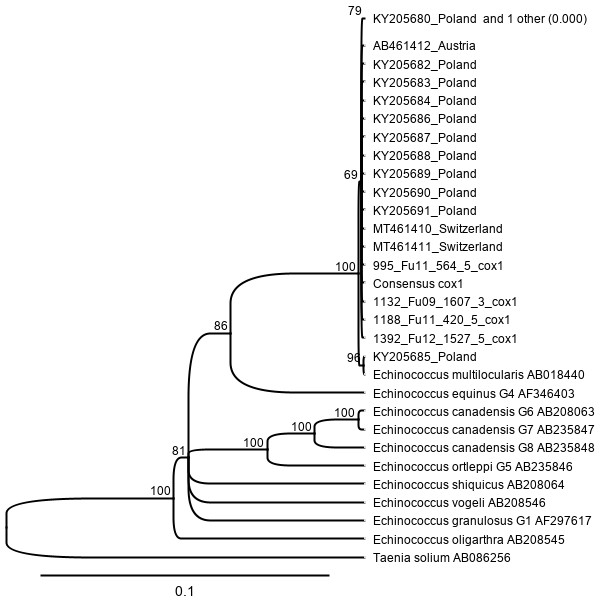


Additional file 1: Figure S3. Dendrogram derived from the *Cox1* gene data of the consensus sequence of all isolates identified in this work, the sequences with the respective SNPs and the sequences taken from Nakao et al. (64). Bootstrap values (%) are shown on the branches.


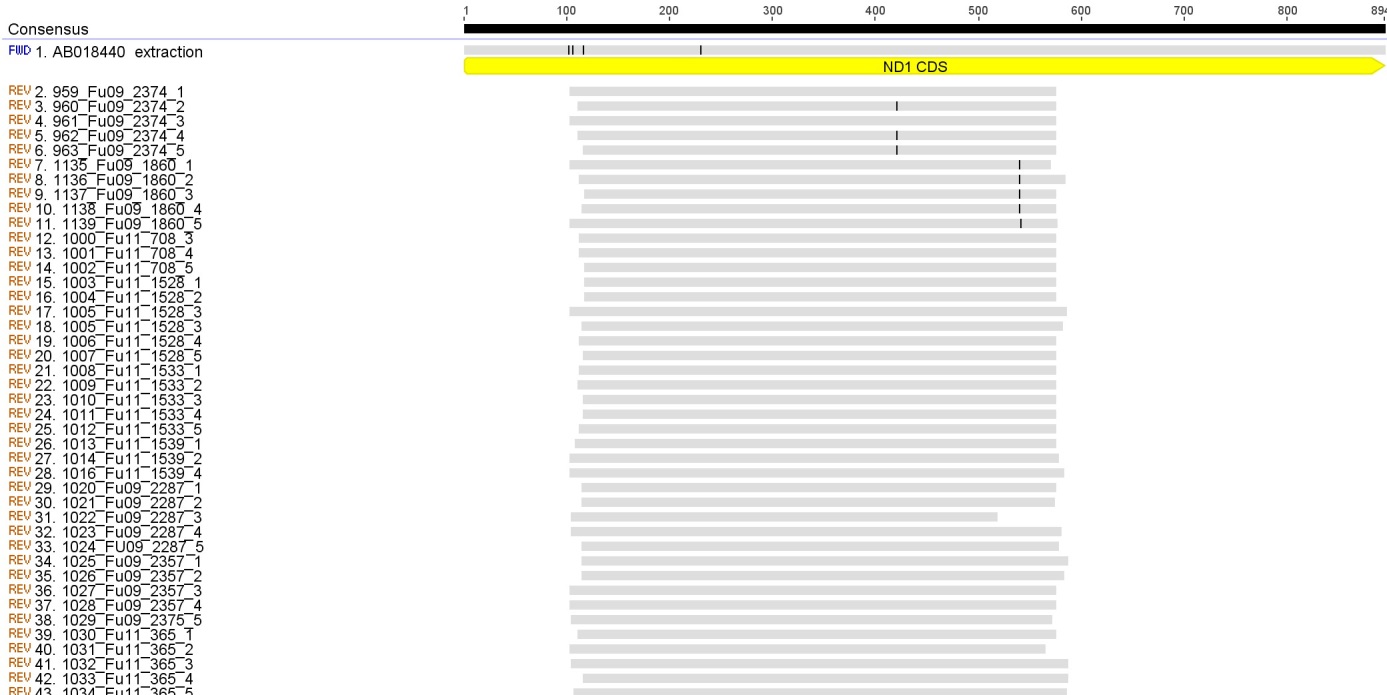


Additional file 1: Figure S4. Alignment of the *Nad1* nucleotide sequences of isolates from Brandenburg and North Rhine-Westphalia and the reference sequence AB018440. The *Nad1* gene is shown (yellow bar). The black bars represent the position of the SNPs in the sequences of the parasite isolates compared to the consensus sequence of all samples investigated in this work.


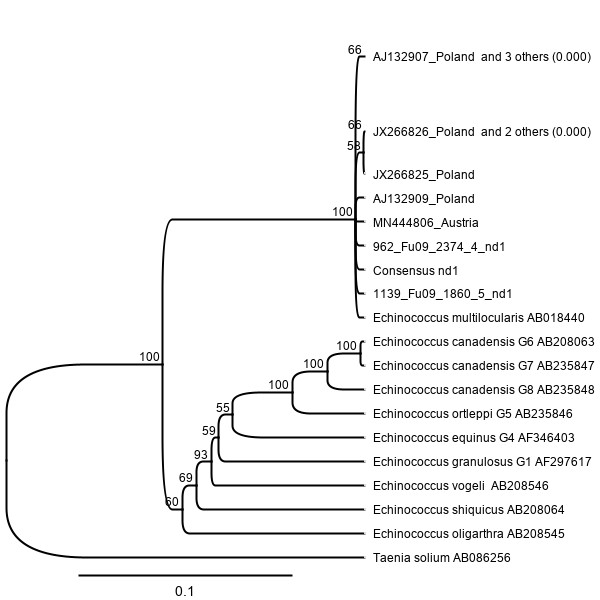


Additional file 1: Figure S5. Dendrogram derived from the *Nad1* gene data of the consensus sequence of all isolates identified in this work, the sequences with the respective SNPs and the sequences taken from Nakao et al. (64). Bootstrap values (%) are shown on the branches.


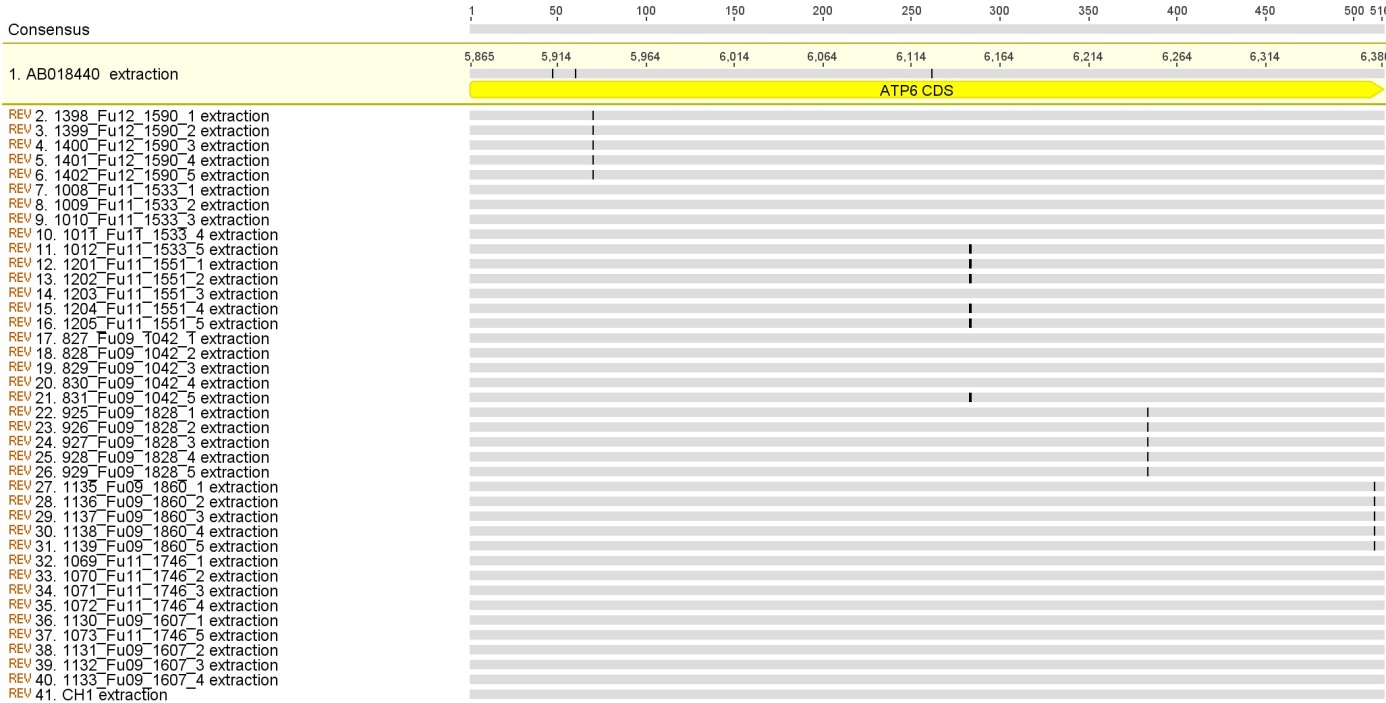


Additional file 1: Figure S6. Alignment of the *ATP6* nucleotide sequences of isolates from Brandenburg and North Rhine-Westphalia and the reference sequence AB018440. The *ATP6* gene is shown (yellow bar). The black bars represent the position of the SNPs in the sequences of the parasite isolates compared to the consensus sequence of all samples investigated in this work.


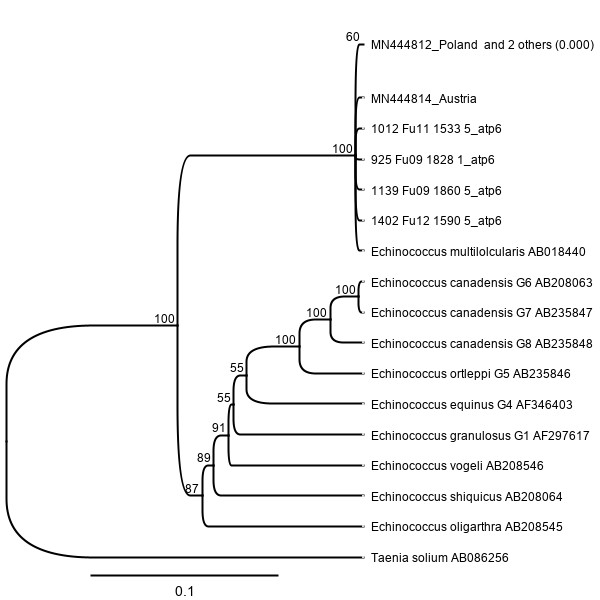


Additional file 1: Figure S7. Dendrogram derived from the atp6 gene data of the consensus sequence of all isolates identified in this work, the sequences with the respective SNPs and the sequences taken from Nakao et al. (64). Bootstrap values (%) are shown on the branches.


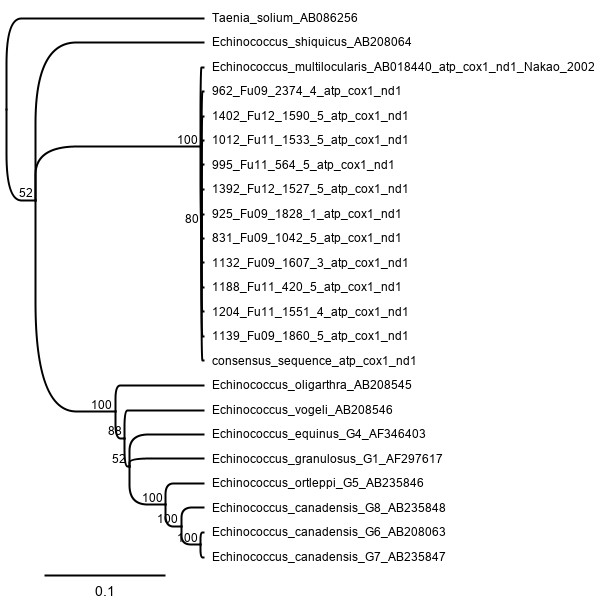


**Additional file 1: Figure S8**. Dendrogram derived from the concatenated (atp6, cox1, nd1) data of the consensus DNA sequences of all isolates identified in this work, the respective SNPs and the sequences taken from Nakao et al. (64). Bootstrap values (%) are shown on the branches


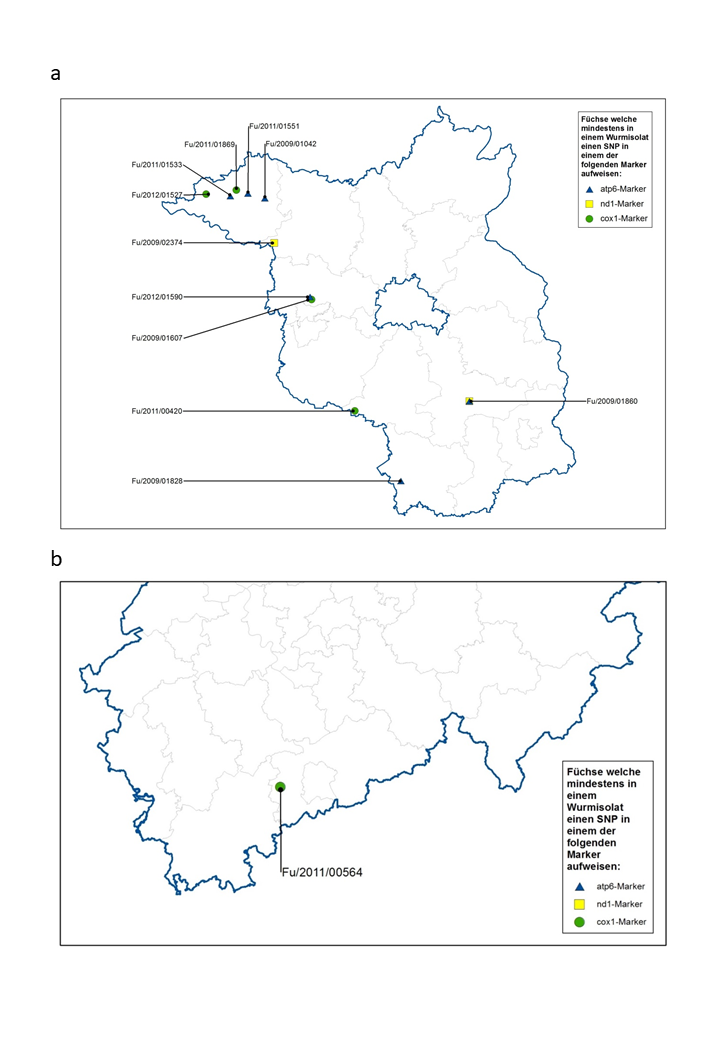


Additional file 1: Figure S9. Geographic representation of the sequence differences (a) in Brandenburg and (b) in North Rhine-Westphalia. The position of foxes with at least one SNP in an isolate of *E. multilocularis* is shown. Blue triangles represent the positions of foxes that had at least one *E. multilocularis* isolate with a SNP in the atp6 gene. Yellow squares represent the positions of foxes with parasites with differences in the *Nad1* gene and the green circles show the positions of foxes that were infected by parasites that had a difference in the *Cox1* gene sequence.
